# LncRNA RASAL2-AS1 serves as a potential biomarker for prognosis and regulates cellular processes via the miR-590-5p/PDCD5 axis and Wnt/β-catenin pathway in cervical cancer

**DOI:** 10.1186/s41065-026-00709-3

**Published:** 2026-07-28

**Authors:** Ke Yin, Yi Li, Mengying Tang

**Affiliations:** 1 Department of Obstetrics and Gynecology, Affiliated Women’s Hospital of Jiangnan University (Wuxi Woman and Enfants Care Hospital), Wuxi, 214000 China; 2 Department of Obstetrics and Gynecology, Songjiang Hospital Affiliated to Shanghai Jiao Tong University School of Medicine, Shanghai, 201699 China; 3 Department of Gynecology, Women’s Hospital of Nanjing Medical University, Nanjing Women and Children’s Healthcare Hospital, No. 123, Tianfei Lane, Mochou Road, Qinhuai District, Nanjing, Jiangsu Province 210004 China

**Keywords:** Cervical cancer, RASAL2-AS1, miR-590-5p, PDCD5, Prognosis

## Abstract

**Background:**

The objective of this research is to determine the relative abundances and clinical significance of the long non-coding RNA (lncRNA) RASAL2-AS1 in cervical cancer. Additionally, this study aims to elucidate its molecular mechanism in influencing malignant progression through the regulation of the microRNA-590-5p (miR-590-5p)/PDCD5 axis and the Wnt/β-catenin signaling pathway.

**Methods:**

Gene expression of RASAL2-AS1 was assessed in cervical cancer tissues (*n* = 103) using quantitative real-time PCR (qRT-PCR). The impacts on cellular events, epithelial-mesenchymal transition (EMT), and key signaling pathways were evaluated using CCK-8, Transwell, and Western Blot techniques. The target relationships among RASAL2-AS1, miR-590-5p, and PDCD5 were verified via luciferase reporter assays.

**Results:**

RASAL2-AS1 exhibited significantly reduced expression in cervical cancer. Its downregulation was markedly associated with advanced FIGO staging (*P* = 0.014), lymph node metastasis (*P* = 0.035), and poor prognosis, establishing it as an independent indicator of unfavorable outcomes for cervical cancer patients (HR = 0.201, 95% CI = 0.087–0.464, *P* < 0.001). Functionally, the upregulation of RASAL2-AS1 inhibited cervical cancer cell growth and EMT. Mechanistically, RASAL2-AS1 acted as a molecular sponge to sequester miR-590-5p, thereby alleviating its inhibitory effect on PDCD5. Furthermore, RASAL2-AS1 obstructs the Wnt/β-catenin pathway through the miR-590-5p/PDCD5 axis.

**Conclusions:**

RASAL2-AS1 exerts a tumor-suppressive effect in cervical cancer by functioning as a competing endogenous RNA (ceRNA) that modulates the miR-590-5p/PDCD5 axis and regulates the Wnt/β-catenin pathway.

## Background

Cervical cancer, as a prevalent malignancy, poses a significant threat to women’s health, with its pathogenesis characterized by a multi-step and multifactorial process [1, 2]. While persistent human papillomavirus (HPV) infection is the primary driver of cervical cancer [3, 4], this infection alone is insufficient to initiate tumorigenesis [5]. Genetic and epigenetic modifications are crucial contributors to the progression of cervical cancer [6]. Consequently, a thorough investigation into the molecular mechanisms underlying cervical carcinogenesis is essential for identifying potential diagnostic biomarkers and therapeutic targets.

Long non-coding RNAs (lncRNAs) are defined as RNA molecules exceeding 200 nucleotides in length that lack protein-coding capability [7]. Recent studies have demonstrated that lncRNAs exhibit abnormal expression in various tumors, including cervical cancer [8, 9]. These transcripts play essential roles in modulating key cellular behaviors such as cell proliferation, differentiation, and metastasis through the regulation of gene expression [10, 11]. Their functions have become a focal point of research in oncology [12]. RASAL2-AS1 is a lncRNA derived from the antisense strand of the RASAL2 gene. Existing studies suggest that RASAL2-AS1 expression exhibits tissue specificity, being highly expressed in head and neck cancers [13], while bioinformatics analyses indicate it may be downregulated in cervical cancer [14]. Nevertheless, the precise expression levels, clinical relevance, and cellular behaviors of RASAL2-AS1 in cervical cancer remain unclear, and its potential mechanisms of action warrant further investigation.

Competing endogenous RNAs (ceRNAs) represent a crucial mechanism through which long non-coding RNAs (lncRNAs) exert their functions. Specifically, lncRNAs can act as molecular sponges that bind and sequester microRNAs (miRNAs), thereby alleviating the repressive effects of miRNAs on their respective target genes. Notably, multiple studies have confirmed that miR-590-5p is significantly elevated in cervical cancer, where it exhibits oncogenic activity [15, 16]. Bioinformatics prediction have revealed potential binding sites between RASAL2-AS1 and miR-590-5p, suggesting a possible ceRNA regulatory relationship between the two. Programmed cell death 5 (PDCD5), a known pro-apoptotic protein, shows downregulated expression in cervical cancer tissues [17]. Further analysis of target gene predictions indicates that PDCD5 may serve as a downstream target of miR-590-5p. Additionally, the dysregulated activation of the Wnt/β-catenin pathway is a critical factor contributing to tumor development and poor prognosis in cervical cancer [18, 19].

In our research, we determined the relative abundances of RASAL2-AS1 in cervical cancer, and assessed its relationships with clinicopathological factors and clinical outcomes. Furthermore, we investigated the effects of RASAL2-AS1 on cellular events in cervical cancer and its role in epithelial-mesenchymal transition (EMT). Additionally, we explored whether the RASAL2-AS1/miR-590-5p/PDCD5 axis influences malignant progression in cervical cancer by modulating the Wnt/β-catenin pathway.

## Materials and methods

### Patient tissue specimens and ethics

This study enrolled 103 cervical cancer patients who underwent surgical resection at the Women’s Hospital of Nanjing Medical University. Tumor tissue specimens and corresponding adjacent noncancerous tissue specimens (collected at least 5 cm from the tumor margin) were obtained from each patient. All tissue specimens were promptly placed in liquid nitrogen for cryopreservation immediately after intraoperative retrieval, and subsequently transferred to a -80℃ ultra-low temperature freezer for storage. All diagnoses were confirmed through postoperative histopathological examination.

All methods were conducted in accordance with relevant guidelines, regulations and the Declaration of Helsinki. The study protocol received ethical approval from the Ethics Committee of the Women’s Hospital of Nanjing Medical University, and written informed consent was obtained from all participants prior to surgery.

Cervical cancer patients were enrolled based on the following criteria: 1) a first-time diagnosis of histopathologically confirmed cervical cancer; 2) no prior neoadjuvant chemotherapy or radiotherapy; and 3) availability of complete clinical, pathological, and follow-up data. Patients were excluded if they met any of the following criteria: 1) a history of concurrent malignant tumors; 2) severe cardiac, hepatic, or renal insufficiency, or autoimmune disease; and 3) incomplete clinical documentation.

### Cell lines and cell culture

The human immortalized normal cervical epithelial cell line H8, along with cervical cancer cell lines SiHa, HeLa, C-33 A, and CaSki were obtained from the American Type Culture Collection (ATCC). All cell lines underwent routine testing for mycoplasma contamination utilizing a PCR-based mycoplasma detection kit (ATCC, Manassas, VA, USA) and were confirmed to be negative. Cell line authentication was conducted through short tandem repeat (STR) profiling (Genesky Biotech, Shanghai), with all profiles aligning with the ATCC reference databases. H8 cells were cultured in RPMI-1640 medium supplemented with 10% fetal bovine serum (FBS), while the cervical cancer cell lines were maintained in high-glucose DMEM medium also supplemented with 10% FBS. All cell lines were incubated at 37 °C in a humidified atmosphere containing 5% CO_2_ and were regularly screened for mycoplasma contamination.

### Cell transfection

Cervical cancer cells were seeded into culture plates and transfected upon reaching 70–80% confluence. Plasmids or oligonucleotides were introduced into the cells using Lipofectamine 3000 according to the manufacturer’s instructions. Overexpression experimental groups included a control (blank control), an overexpression negative control (oe-NC), and oe-RASAL2-AS1. Functional rescue experiments were categorized into: oe-NC + mimic-NC, oe-RASAL2-AS1, oe-RASAL2-AS1 + miR-590-5p mimic. The pathway rescue experiments were organized as follows: oe-RASAL2-AS1 + mimic-NC, oe-RASAL2-AS1 + miR-590-5p, oe-RASAL2-AS1 + si-NC, oe-RASAL2-AS1 + si-PDCD5.

### RNA isolation and real-time quantitative PCR (qRT-PCR)

Total RNA was extracted using TRIzol reagent (Life Technology, Waltham, MA, USA). The RNA concentration was assessed utilizing a microvolume spectrophotometer (NanoDrop 2000, USA). All samples exhibited an A260/280 ratio maintained within the range of 1.8 to 2.1. cDNA synthesis was performed according to the protocol provided by the PrimeScript RT Reagent Kit (TaKaRa, Japan). Amplification reactions were conducted with SYBR Premix Ex TaqII (TaKaRa, Dalian, China) on a QuantStudio 5 real-time quantitative PCR system (Thermo Fisher Scientific, USA). GAPDH was employed as the internal reference gene for RASAL2-AS1 and PDCD5, while U6 was used for miR-590-5p. Relative expression levels were quantified using the 2^−ΔΔCt^ method, with each experiment conducted in triplicate.

### Cell proliferation detection

Cervical cancer cells subjected to the corresponding transfection treatments were uniformly seeded into a 96-well plate at a density of 3 × 10^3^ per well. Following the seeding, the cell plates were incubated at 37℃ with 5%CO_2_ for 24, 48, 72, and 96 h respectively. At each predetermined time point, 10µL of CCK-8 solution (Tohoku Chemical Laboratory, Japan) was added to each well, followed by an additional 2-hour incubation. The absorbance values at 450 nm were measured for each well using a microplate reader.

### Transwell assay

For migration assays, serum-free cell suspensions were directly added to the upper chamber. In contrast, for invasion assays, the upper chamber was pre-coated with Matrigel matrix gel (BD Biosciences) at 37 °C for one hour to simulate the basement membrane. Medium supplemented with 20% FBS, serving as a chemoattractant, was added to the lower chamber. After incubation at 37 °C for 48 h, cells were fixed in 4% paraformaldehyde and subsequently stained with 0.1% crystal violet. Non-migrated cells on the upper surface were gently removed using a cotton swab. Migrated cells were then counted from selected fields using an inverted microscope.

### Western blot assays

Following extraction with RIPA lysis buffer and quantification via BCA, equal protein loads were separated using 10% SDS-PAGE and transferred to PVDF membranes in a wet transfer apparatus. Membranes were blocked with 5% skimmed milk at room temperature for one hour, followed by overnight incubation with specific primary antibodies at 4 °C (Cell Signaling Technology). After washing with TBST, incubation with HRP-labelled secondary antibody at room temperature for one hour was performed. Finally, detection was achieved using ECL chemiluminescent substrate on a chemiluminescence imaging system. Protein loading quantities were normalized using GAPDH as the internal control.

### Dual luciferase reporter assay

Binding sites between RASAL2-AS1 and miR-590-5p, as well as between miR-590-5p and the PDCD5 3’ UTR, were predicted using bioinformatics platforms such as StarBase. Sequences of RASAL2-AS1 or PDCD5 3’UTR containing wild-type (WT) or mutant (MUT) binding sites were cloned into the pGL3-Basic luciferase reporter vector. The reporter vector was co‑transfected with miR‑590‑5p mimic or mimic NC into cells. Luciferase activity was evaluated 48 h after transfection using a dual‑luciferase assay and normalized to Renilla luciferase activity as an endogenous reference.

### Statistics

Data analysis was conducted using SPSS version 26.0 and GraphPad Prism version 9.0. Continuous variables are presented as mean ± standard deviation (SD). Two-group comparisons were performed using Student’s t-test, while multi-group comparisons were conducted using one-way ANOVA. Categorical data are reported as counts and percentages, and were compared using the chi-square test or Fisher’s exact test, as appropriate. Survival curves were generated using the Kaplan–Meier method and compared via the log-rank test. Both univariate and multivariate survival analyses were executed using Cox proportional hazards regression models. A *P* value of less than 0.05 was deemed statistically significant.

## Results

### RASAL2-AS1 is decreased in cervical cancer

**Fig. 1:**
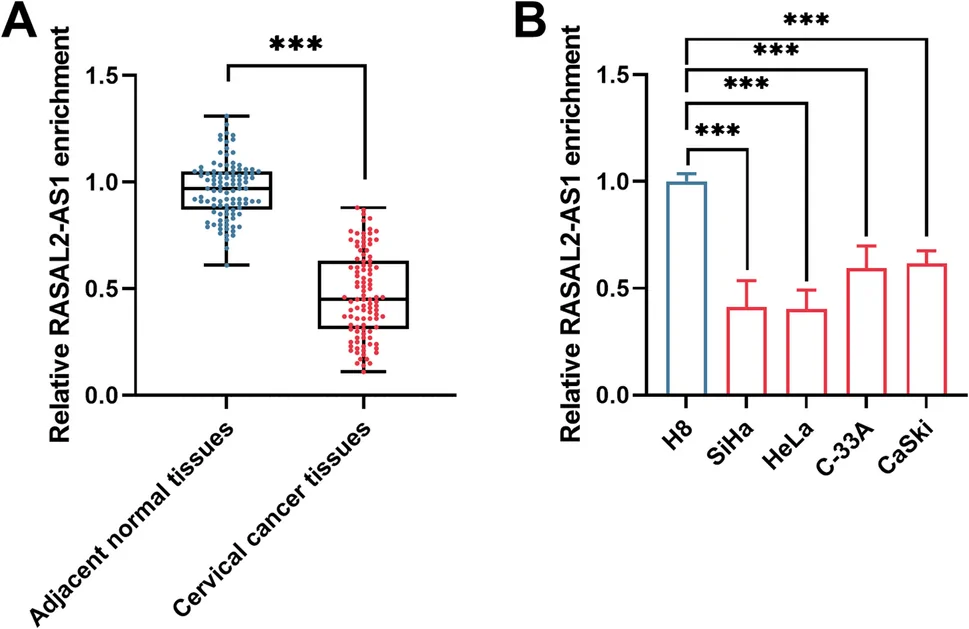
RASAL2-AS1 is downregulated in cervical cancer. **A**. Relative expression levels of RASAL2-AS1 in cervical cancer tissues. Data are shown as mean ± standard deviation (SD). **B**. Relative expression of RASAL2-AS1 in human normal cervical epithelial cell line H8 and cervical cancer cell lines (SiHa, HeLa, C-33 A, and CaSki)

The qRT-PCR analysis revealed a significant reduction in the relative abundance of RASAL2-AS1 in cervical cancer tissue specimens compared to matched normal tissue samples (*P* < 0.001, Fig. 1A). Additionally, RASAL2-AS1 expression was markedly diminished in several cervical cancer cell lines (SiHa, HeLa, C-33 A, CaSki) when compared to human normal cervical epithelial cells H8 (*P* < 0.001, Fig. 1B).

### RASAL2-AS1 correlates with clinical pathological parameters of cervical cancer patients

Using the mean expression level of RASAL2‑AS1 as the cutoff, the 103 cervical cancer patients were stratified into a low‑expression group (*n* = 57) and a high‑expression group (*n* = 46). Analysis of clinicopathological characteristics (Table 1) showed that low RASAL2‑AS1 expression was significantly associated with late FIGO stage (III‑IV, *P* = 0.014) and lymph node metastasis positivity (*P* = 0.035). On the other hand, no obvious association was observed of RASAL2‑AS1 expression with patient age (*P* = 0.616), tumor size (*P* = 0.090), or histological type (*P* = 0.897).

**Table 1:** Association of RASAL2-AS1 expression with clinicopathological features of cervical cancer patients

Features	Cases (*n* = 103)	RASAL2-AS1 expression		*P* value
		Low (*n* = 57)	High (*n* = 46)	
Age (years)				0.616
< 40	42	22	20	
≥ 40	61	35	26	
Tumor size (cm)				0.090
< 4	67	33	34	
≥ 4	36	24	12	
FIGO stage				0.014
I-II	75	36	39	
III-IV	28	21	7	
Histology type				0.897
Squamous cell cancer	80	44	36	
Adenocarcinoma	23	13	10	
Lymph node metastasis				
Negative	85	43	42	0.035
Positive	18	14	4	

*P* < 0.05 indicates a statistical difference

*FIGO* the International Federation of Gynecology and Obstetrics

### Low RASAL2-AS1 expression predicts the unfavorable prognosis of cervical cancer

**Fig. 2:**
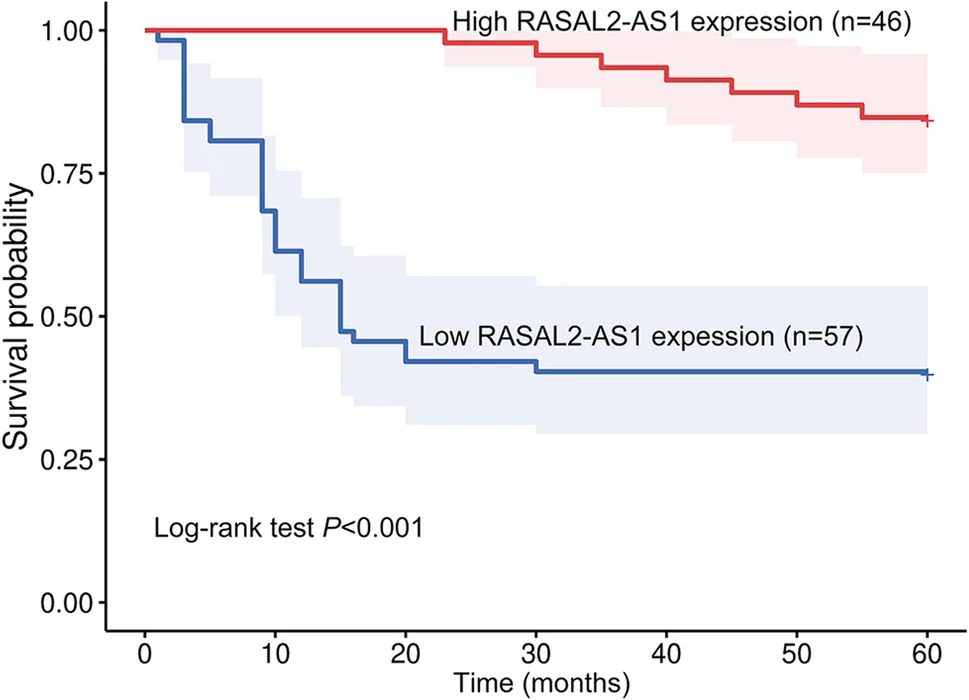
Low RASAL2-AS1 expression predicts unfavorable clinical outcome in cervical cancer. Kaplan-Meier survival curves were plotted and a statistical comparison was performed using the log-rank test

Survival analysis evaluated the prognostic significance of RASAL2-AS1 expression, indicating that patients with low RASAL2-AS1 expression exhibited significantly poorer overall survival compared to those with high expression levels (*P* < 0.001, Fig. 2). Cox regression analysis further confirmed its independent prognostic predictive value (Table 2). Univariate analysis revealed a significant association between low RASAL2-AS1 expression and poor prognosis (HR = 0.164, 95% CI: 0.072–0.373, *P* < 0.001). After adjusting for clinical and pathological factors, including FIGO stage and lymph node metastasis in the multivariate analysis, low RASAL2-AS1 expression remained an independent index of unfavorable patient prognosis (HR = 0.201, 95% CI: 0.087–0.464, *P* < 0.001).

**Table 2:** Cox regression analysis of clinicopathological features for overall survival in cervical cancer patients

Features	Univariate analysis		Multivariate analysis	
	HR (95%CI)	*P* value	HR (95%CI)	*P* value
Age (years)	0.650 (0.352–1.199)	0.168	/	/
Tumor size (cm)	1.685 (0.911–3.116)	0.096	/	/
FIGO stage	2.889 (1.552–5.376)	0.001	2.228 (1.180–4.204)	0.013
Histology type	1.264 (0.603–2.649)	0.535	/	/
Lymph node metastasis	3.234 (1.663–6.288)	0.001	2.563 (1.293–5.078)	0.007
RASAL2-AS1	0.164 (0.072–0.373)	< 0.001	0.201 (0.087–0.464)	< 0.001

*P* < 0.05 indicates a statistical difference

*FIGO* the International Federation of Gynecology and Obstetrics, *HR* Hazard Ratio, *95%CI* 95% confidence interval

### RASAL2-AS1 overexpression suppresses cervical cancer cellular behaviors and EMT

**Fig. 3:**
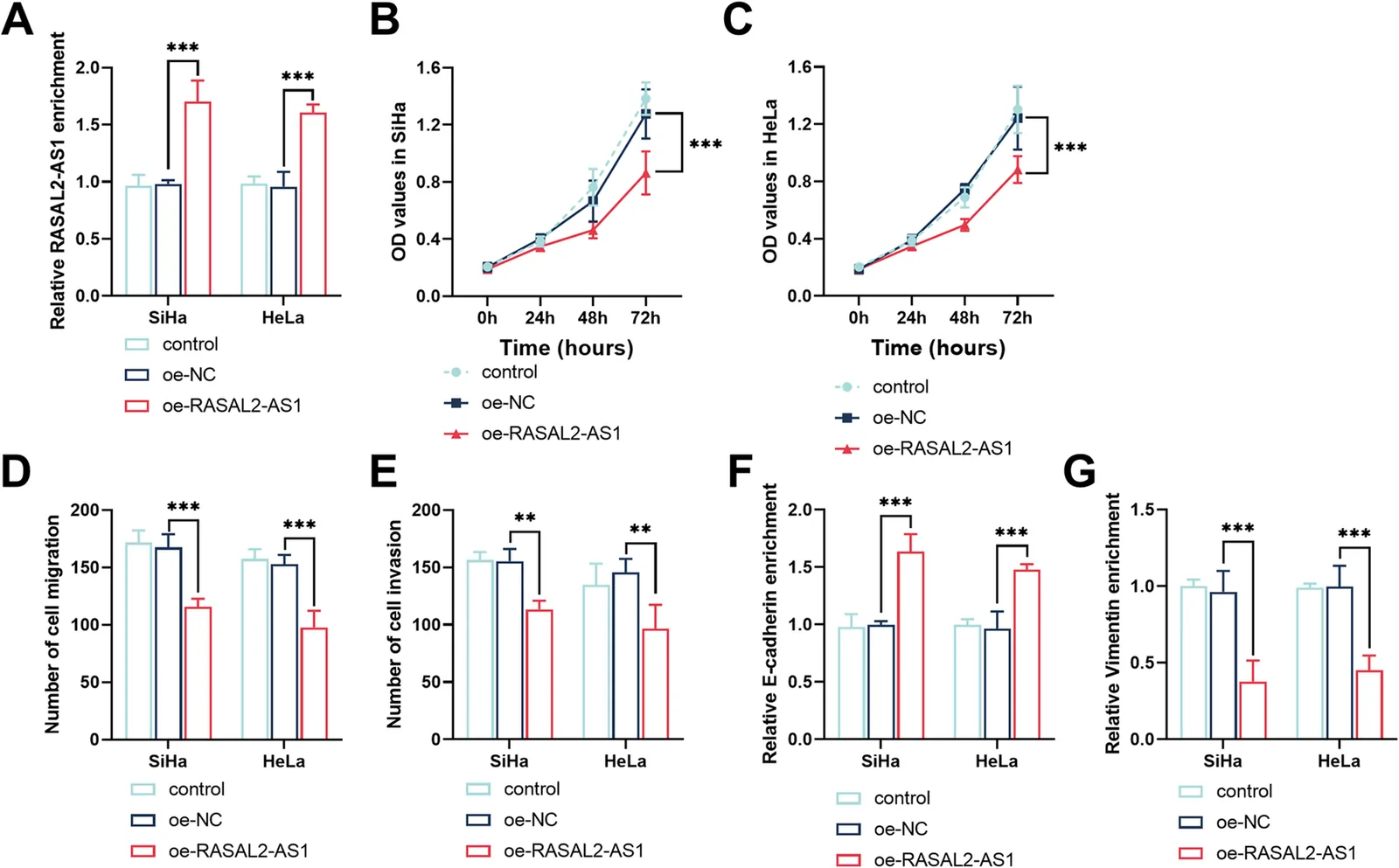
Overexpression of RASAL2-AS1 suppresses cervical cancer cell growth and epithelial-mesenchymal transition (EMT). **A**. The efficiency of RASAL2-AS1 overexpression was validated in cervical cancer cells transfected with oe-RASAL2-AS1. **B-C**. Cell proliferation was assessed in SiHa (**B**) and HeLa (**C**) cells after RASAL2-AS1 overexpression at indicated time points. **D-E**. the cell number of migration (**D**) and invasion (**E**) was analyzed in SiHa and HeLa cells. **F-G**. E-cadherin (**F**) and Vimentin (**G**) expressions were compared in SiHa and HeLa cells after RASAL2-AS1 overexpression

To evaluate the cellular function of RASAL2-AS1 in cervical cancer, overexpression models were established in SiHa and HeLa cells. qRT-PCR confirmed a significantly enhanced transfection efficiency (Fig. 3A). Compared to the control and oe-NC groups, the proliferation capacity of oe-RASAL2-AS1 cells was significantly suppressed at 48, 72, and 96 h (Fig. 3B-C). Transwell migration and invasion assays further demonstrated that both cell lines exhibited markedly reduced migration and invasion following RASAL2-AS1 overexpression (Fig. 3D-E). Additionally, qRT-PCR analysis revealed an upregulation of E-cadherin in the oe-RASAL2-AS1 group (Fig. 3F), and a downregulation of Vimentin (Fig. 3G).

### A target interaction of RASAL2-AS1 with miR-590-5p expression

**Fig. 4:**
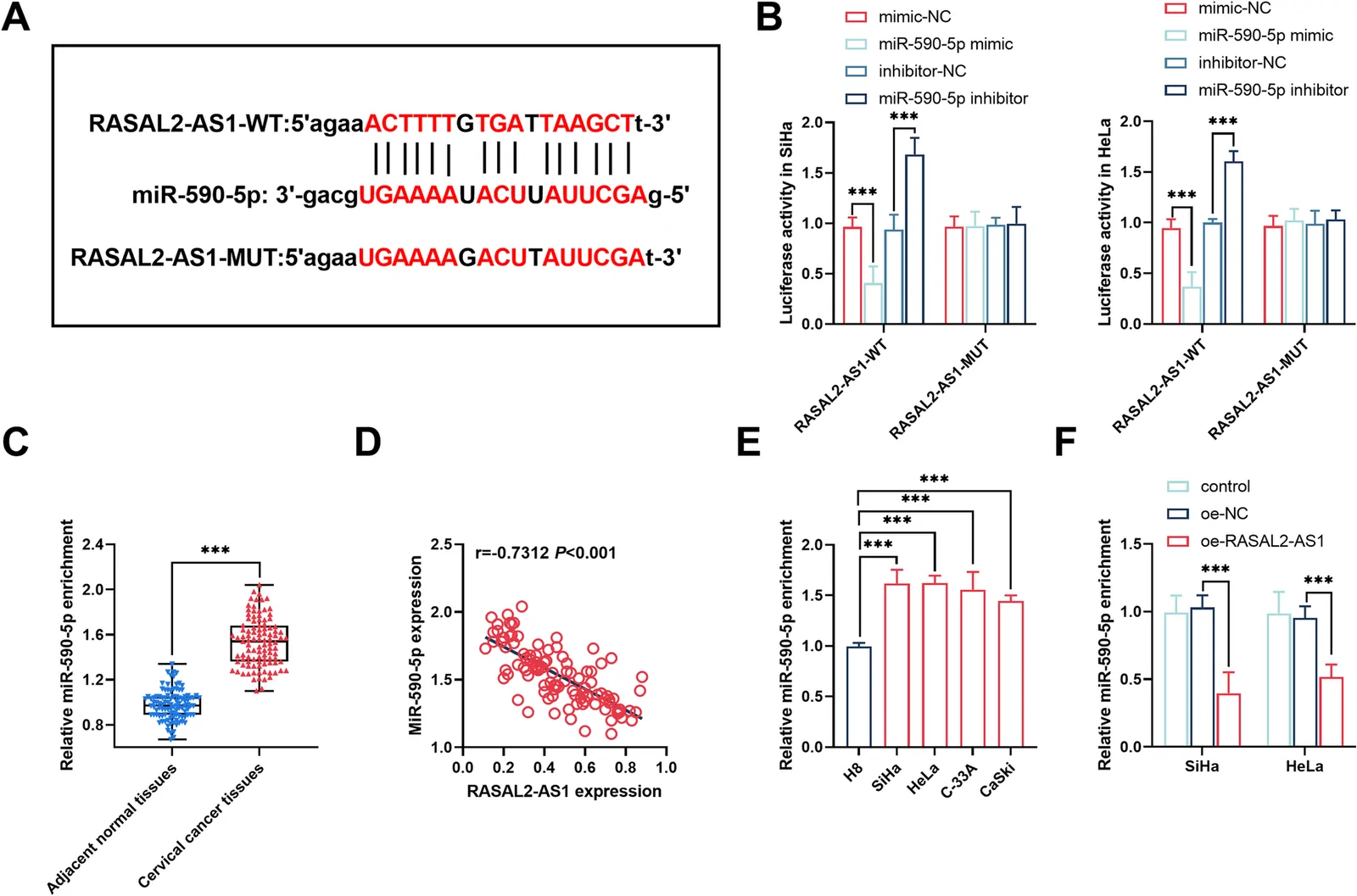
RASAL2-AS1 directly targets and inhibits miR-590-5p. **A**. A schematic diagram showing the predicted binding sites between RASAL2-AS1 and miR-590-5p is presented. **B**. A dual-luciferase reporter assay was performed in SiHa and HeLa cells co-transfected with RASAL2-AS1-WT or RASAL2-AS1-MUT reporter plasmids, as well as with miR-590-5p mimic/inhibitor. **C**. Relative expression of miR-590-5p in tissue samples from cervical cancer patients. **D**. Correlation analysis of RASAL2-AS1 and miR-590-5p expression levels in clinical samples (*n* = 103, *r* = -0.7312, *P* < 0.001). **E**. Relative abundances of miR-590-5p in cervical cancer cell lines. **F**. Relative abundances of miR-590-5p in cervical cells after RASAL2-AS1 overexpression transfection

To validate the interaction between RASAL2-AS1 and miR-590-5p, we initially predicted a binding site for the two using bioinformatics analysis (Fig. 4A). Dual luciferase reporter assays indicated that in SiHa and HeLa cells, increased expression of miR-590-5p significantly suppressed luciferase activity in the wild-type RASAL2-AS1 reporter construct, while having no effect on the mutant variant (Fig. 4B). Analysis of clinical sample revealed that miR-590-5p levels were markedly elevated in cervical cancer tissues (*P* < 0.001, Fig. 4C). Correlation analysis demonstrated a significant negative correlation between RASAL2-AS1 and miR-590-5p expression (*r* = -0.7312, *P* < 0.001; Fig. 4D). Expression levels were also elevated in cancer cell lines (*P* < 0.001, Fig. 4E). Furthermore, overexpression of RASAL2-AS1 significantly reduced miR-590-5p levels in SiHa and HeLa cells (*P* < 0.001, Fig. 4F).

### MiR-590-5p overexpression reverses the inhibitory effects of RASAL2-AS1 overexpression in cervical cancer cellular events and EMT

**Fig. 5:**
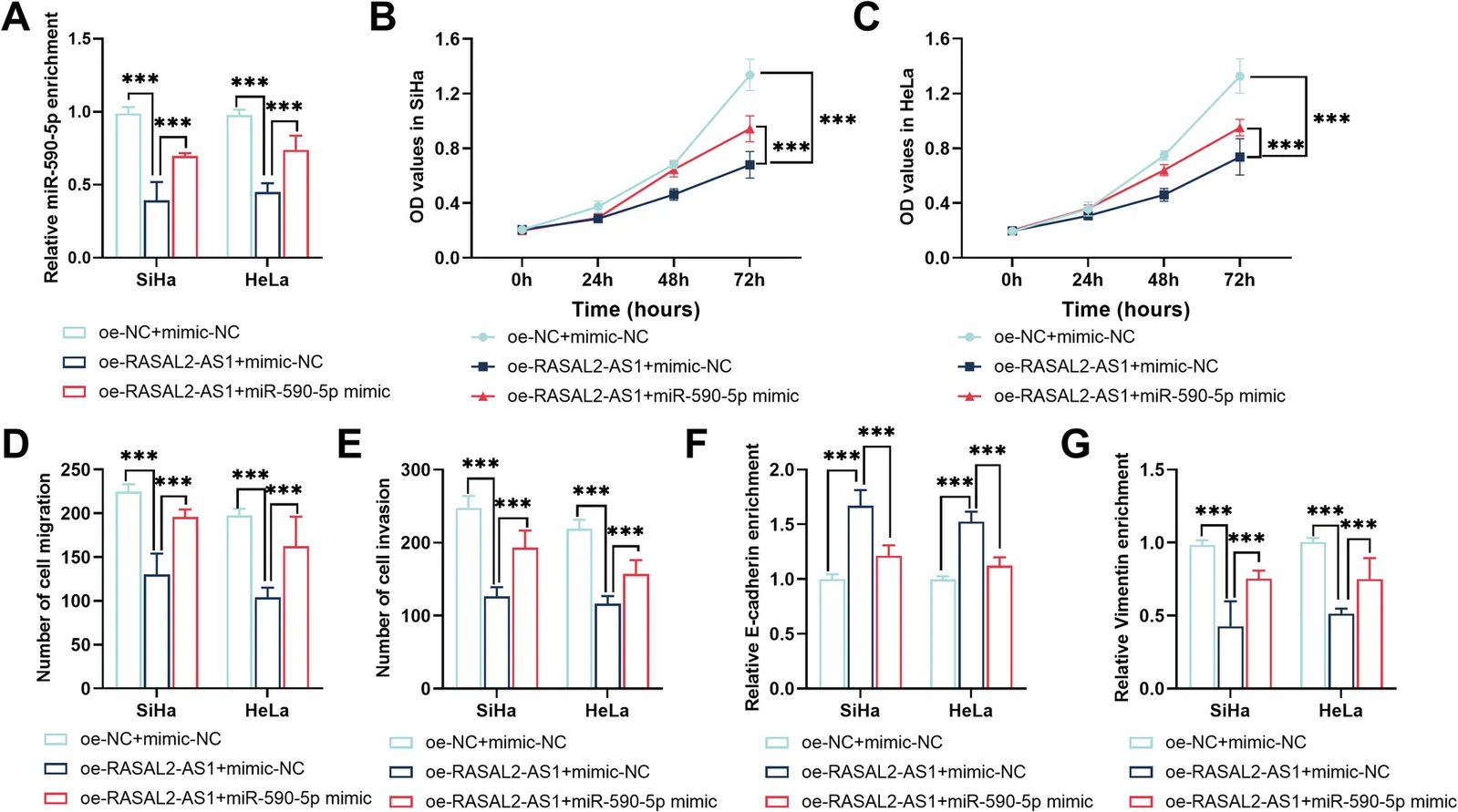
Restoration of miR-590-5p expression counteracts the tumor-suppressive function of RASAL2-AS1. **A**. The expression levels of RASAL2-AS1 and miR-590-5p were validated in cervical cancer cells under different transfection conditions. **B-C**. Cell proliferation was assessed in cervical cancer cells. **D-E**. Transwell migration (**D**) and invasion (**E**) assays. **F-G**. E-cadherin (**F**) and Vimentin (**G**) mRNA expression levels were assessed

To validate whether RASAL2-AS1 exerts its tumor-suppressing effect by targeting miR-590-5p, we conducted a functional recovery experiment. The results demonstrated that co-transfection of a miR-590-5p mimic with oe-RASAL2-AS1 effectively restored miR-590-5p expression levels (*P* < 0.001, Fig. 5A). This enhancement in miR-590-5p expression significantly reversed the proliferation inhibition mediated by RASAL2-AS1 in SiHa and HeLa cells (*P* < 0.001, Fig. 5B-C). Concurrently, overexpression of miR-590-5p effectively restored cellular migration and invasion capabilities (*P* < 0.001, Fig. 5D-E). Furthermore, miR-590-5p overexpression reversed the suppression of the EMT process induced by RASAL2-AS1, as evidenced by reduced E-cadherin expression and elevated Vimentin expression (*P* < 0.001, Fig. 5F-G).

### MiR-590-5p targets and inhibits PDCD5 transcription

**Fig. 6:**
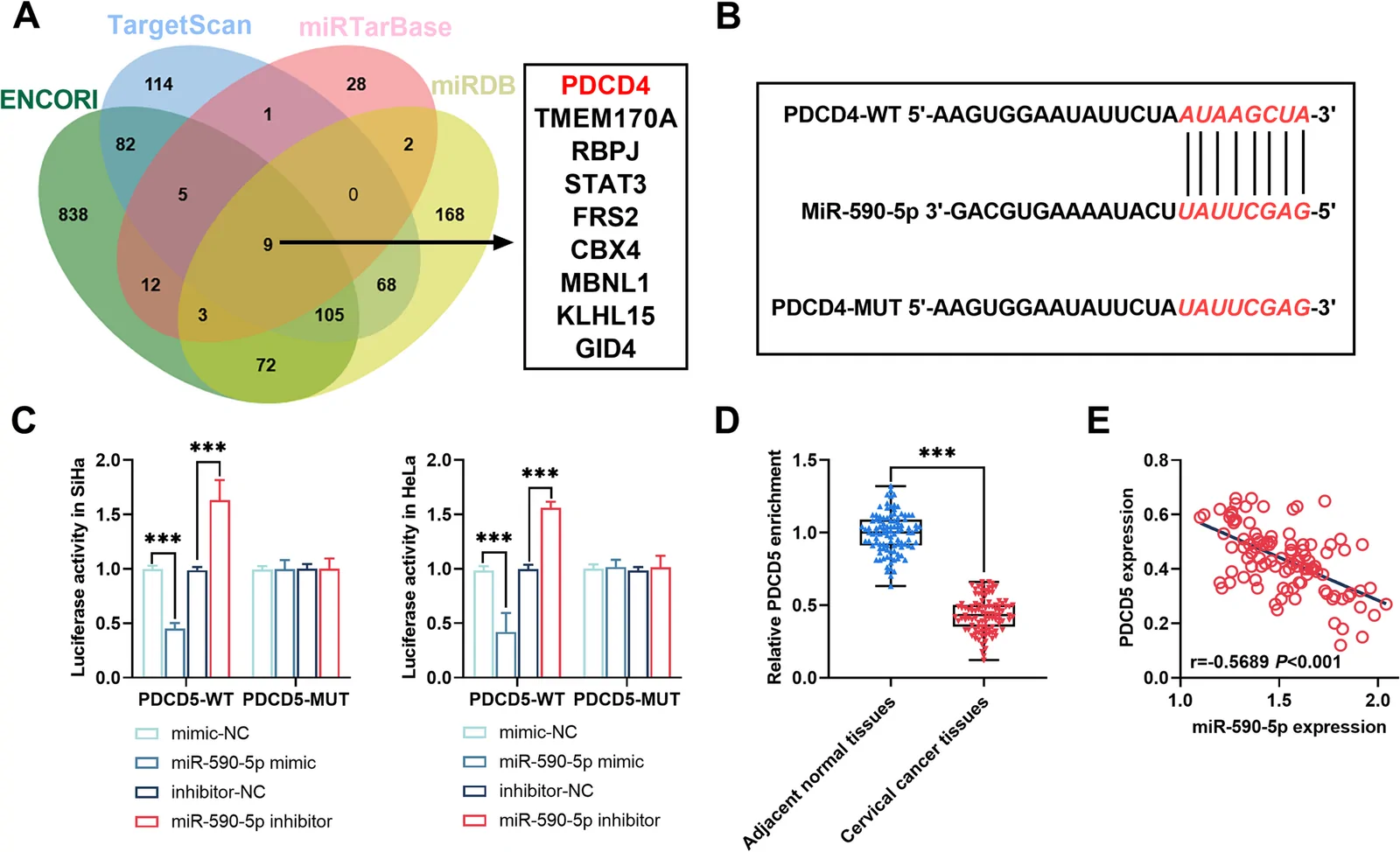
MiR-590-5p directly targets and suppresses PDCD5. **A**. Venn diagram showing the predicted target genes of miR-590-5p that overlap across multiple databases. PDCD5 is identified as a common candidate. **B**. Schematic representation of the predicted binding sites of miR-590-5p with the wild-type (WT) or mutant (MUT) 3’UTR of PDCD5. **C**. A dual-luciferase reporter assay was performed in cervical cancer cells that had been co-transfected with PDCD5 3’UTR-WT or -MUT reporter plasmids along with miR-590-5p mimic or inhibitor, and their respective controls. **D**. Relative expression level of PDCD5 in cervical cancer tissues. **E**. Inverse correlation analysis of miR-590-5p and PDCD5 expression levels in cervical cancer tissues (*n* = 103, *r*=-0.5689, *P* < 0.001)

To investigate the downstream regulatory mechanism of miR-590-5p, potential target genes were identified through bioinformatics analysis, with PDCD5 identified as a candidate gene due to its significant role in tumorigenesis (Fig. 6A). Sequence analysis revealed complementary binding sites of miR-590-5p within the PDCD5 3’UTR (Fig. 6B). Dual luciferase reporter assays demonstrated that the miR-590-5p mimic significantly inhibited luciferase activity in wild-type PDCD5 reporter vectors, while no changes were observed in mutant vectors. Conversely, the miR-590-5p inhibitor produced a contrasting effect, further supporting its functional role (*P* < 0.001, Fig. 6C). Analysis of clinical sample analysis revealed that PDCD5 was significantly downregulated in cervical cancer tissue samples (*P* < 0.001, Fig. 6D). Furthermore, a significant inverse relationship was established between miR-590-5p and PDCD5 expression (*r* = -0.5689, *P* < 0.001, Fig. 6E).

### RASAL2-AS1 regulates the miR-590-5p/PDCD5 axis to repress Wnt/β-catenin pathway

**Fig. 7:**
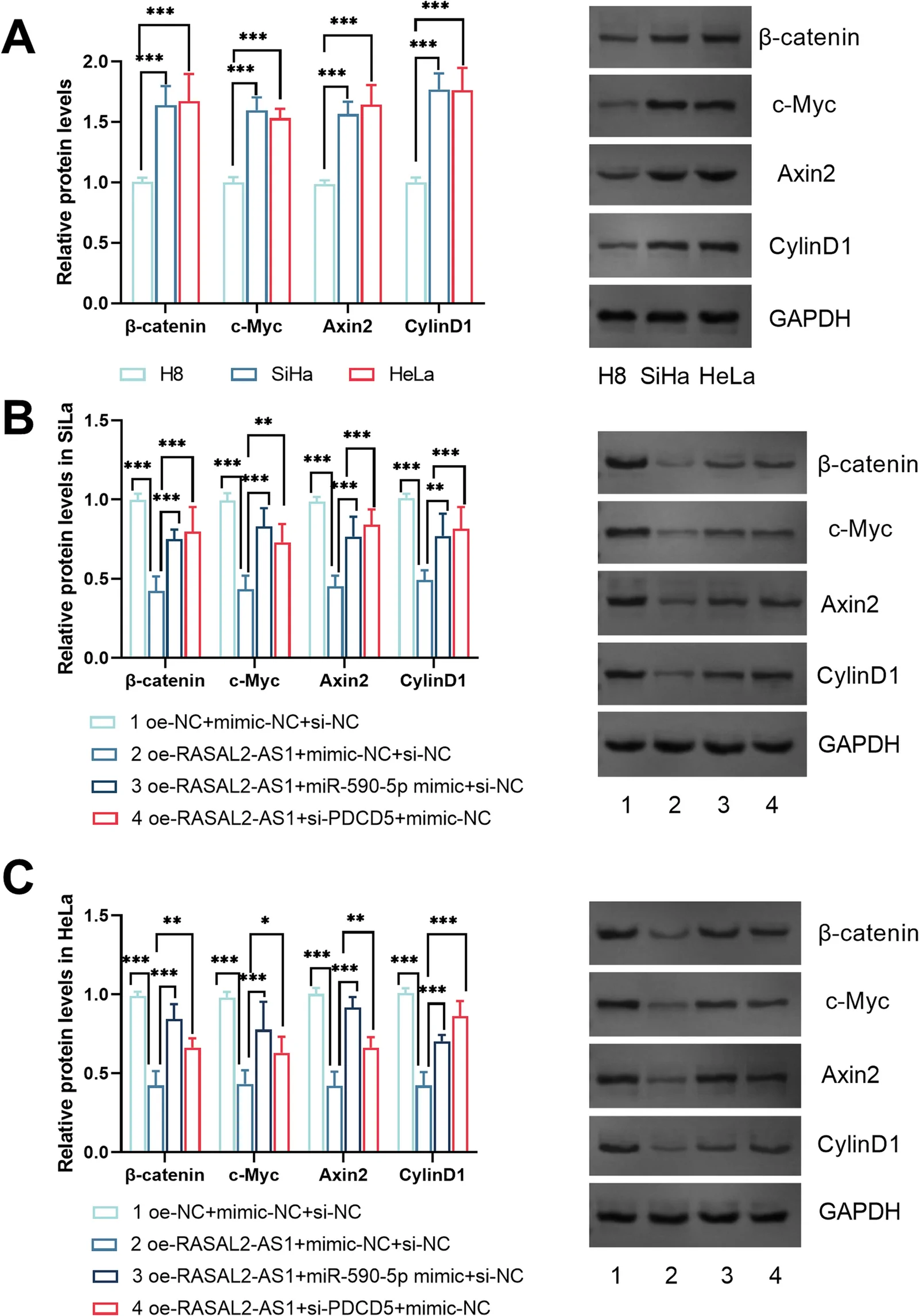
RASAL2-AS1 suppresses the Wnt/β-catenin pathway via the miR-590-5p/PDCD5 axis. **A**. Western blot analysis of key proteins in the Wnt/β-catenin pathway, including β-catenin, c-Myc, Axin2, and CyclinD1, in H8, SiHa and HeLa cells. **B-C**. Western blot analysis of the Wnt/β-catenin pathway proteins in SiHa (**B**) and HeLa (**C**) cells under different treatment conditions

To explore the molecular pathways regulated by RASAL2-AS1, we examined several related proteins associated with the Wnt/β-catenin signaling pathway. Elevated expressions of β-catenin, c-Myc, Axin2, and Cyclin D1 were observed in tumor cell lines (*P* < 0.001, Fig. 7A). The enrichment of RASAL2-AS1 significantly inhibited THE protein levels of β-catenin, Axin2, and Cyclin D1 in cervical cancer cells (*P* < 0.001, Fig. 7B-C). Furthermore, reversal experiments demonstrated that co-transfection of miR-590-5p mimic or si-PDCD5 with the overexpressed RASAL2-AS1 vector effectively reversed the suppressive effects on the Wnt/β-catenin pathway induced byRASAL2-AS1 overexpression (*P* < 0.001, Fig. 7B-C). Notably, the overexpression of miR-590-5p overexpression or the knockdown of PDCD5 significantly restored the levels of several proteins, including β-catenin, c-Myc, Axin2, and CyclinD1.

## Discussion

This study systematically elucidates the tumor-suppressing role of RASAL2-AS1 in cervical cancer. Our results demonstrate that RASAL2-AS1 is obviously decreased across patient tissues and various cervical cancer cell lines. Importantly, low RASAL2-AS1 expression is closely associated with key indicators of tumor aggressiveness, including advanced FIGO stage and lymph node metastasis, and serves as an independent indicator of unfavorable patient prognosis, which aligns with the protective HR < 1 observed in Cox regression. These consistent clinical and molecular findings strongly support the identification of RASAL2-AS1 as a critical tumor inhibitor in the pathogenesis of cervical cancer.

The biological functions of lncRNAs exhibit high tissue specificity. Interestingly, RASAL2-AS1, which is downregulated and functions as a tumor inhibitor in cervical cancer in this study, has been reported as an oncogene that is highly expressed and promotes tumor formation in head and neck squamous cell carcinoma (HNSCC) [13]. The functional heterogeneity of RASAL2-AS1 between HNSCC and cervical cancer may arise from several mechanisms. First, the ceRNA network is highly tissue-specific. MiR-590-5p is oncogenic in cervical cancer but may be downregulated or functionally altered in HNSCC [20], leading to apposing effects of RASAL2-AS1. Second, RASAL2-AS1 may exert its effects through different miRNAs. In addition to miR-590-5p, bioinformatics predictions suggest it may also bind to miR-21, the miR-200 family and others. Differences in the expression levels and functional orientations of these miRNAs across different tumors [21] may contribute to the functional heterogeneity of RASAL1-AS1. Third, tissue-specific transcription factors may drive divergent RASAL2-AS1 expression in different tumors. Future studies employing cross-cancer multi-omics comparisons (e.g., RNA-seq, miRNA-seq, ChIp-seq) are warranted to dissect these mechanisms.

Through a series of functional experiments, we demonstrated that the overexpression of RASAL2-AS1 effectively inhibits cellular behaviors and EMT processes in cervical cancer cells. To further elucidate the underlying mechanisms, we focused on the classical ceRNA regulatory pathway. Our findings indicate that RASAL2-AS1 directly binds to miR-590-5p. Both this study and previous literature confirm a significant increase in miR-590-5p levels in cervical cancer [15, 16]. A significant negative correlation was observed between RASAL2-AS1 and miR-590-5p expression in clinical specimens, with RASAL2-AS1 overexpression effectively reducing intracellular miR-590-5p levels. More importantly, functional rescue experiments demonstrated that re-overexpressing miR-590-5p reversed the suppression of malignant phenotypes in tumor cells mediated by RASAL2-AS1. These results confirm that RASAL2-AS1 exerts its tumor-suppressing function, at least partially, through its interaction with miR-590-5p.

To further refine this regulatory axis, downstream targets of miR-590-5p were predicted and verified. PDCD5, a known pro-apoptotic protein [22], emerged as a candidate and was confirmed to be a direct target, underscoring its functional significance. This finding aligns closely with previous studies demonstrating the downregulation of PDCD5 in cervical cancer and renal cell carcinoma [17, 23]. Our experiments confirmed that the miR-590-5p mimic suppresses PDCD5 expression, revealing a negative correlation between the two in cervical cancer tissues. Consequently, we propose a complete RASAL2-AS1/miR-590-5p/PDCD5 regulatory axis.

Dysregulation of the Wnt/β-catenin signaling pathway is widely implicated in tumorigenesis and contributes significantly contributes to the formation of cervical cancer, among other malignancies [24–26]. This study reveals that RASAL2-AS1 overexpression markedly suppresses the protein levels of β-catenin and its downstream target genes. Moreover, either overexpressing miR-590-5p or knocking down PDCD5 effectively reverses the inhibitory effect of RASAL2-AS1 on the Wnt/β-catenin pathway. Our findings confirm that RASAL2-AS1-mediated regulation of the miR-590-5p/PDCD5 axis leads to the suppression of the Wnt/β-catenin pathway, providing a novel mechanistic perspective on its upstream modulation in cervical carcinogenesis.

While the findings are encouraging, several limitations warrant consideration. Firstly, the sample size, although clinically meaningful, remains moderate. Larger multicenter cohorts are necessary to further validate the prognostic value of RASAL2-AS1 across diverse populations. Secondly, although we established a RASAL2-AS1/miR-590-5p/PDCD5/Wnt/β-catenin axis in vitro, in vivo validation using animal models is essential to confirm its physiological relevance and therapeutic potential. Thirdly, high-risk HPV infection, particularly HPV16 and HPV18, constitutes a significant etiological factor in cervical carcinogenesis [27, 28]. Our study did not stratify patients based on HPV status or investigate whether RASAL2-AS1 expression is influenced by HPV oncoproteins (E6/E7), which represents an important direction for future research. Furthermore, as a ceRNA, RASAL2-AS1 may interact with other miRNAs beyond miR-590-5p, and PDCD5 may not be its sole functional downstream effector. A more comprehensive analysis using techniques such as RNA pulldown combined with sequencing could identify additional binding partners and provide a more holistic view of its regulatory network.

In summary, this study identifies RASAL2‑AS1 as a novel tumor‑suppressive lncRNA in cervical cancer. By competitively binding to miR‑590‑5p, RASAL2‑AS1 elevates PDCD5 expression, which in turn inhibits the oncogenic Wnt/β‑catenin pathway and restrains malignant progression. These results advance the molecular understanding of cervical cancer pathogenesis and offer a theoretical foundation for developing prognostic biomarkers and therapeutic approaches centered on the RASAL2‑AS1 pathway. Future research may focus on developing therapies targeting the restoration of RASAL2-AS1 expression and exploring its potential for combination use with existing treatments.

## Data Availability

The datasets used and/or analysed during the current study are available from the corresponding author on reasonable request.
